# A DNA damage repair gene‐associated signature predicts responses of patients with advanced soft‐tissue sarcoma to treatment with trabectedin

**DOI:** 10.1002/1878-0261.12996

**Published:** 2021-06-30

**Authors:** David S. Moura, Maria Peña‐Chilet, Juan Antonio Cordero Varela, Ramiro Alvarez‐Alegret, Carolina Agra‐Pujol, Francisco Izquierdo, Rafael Ramos, Luis Ortega‐Medina, Francisco Martin‐Davila, Carolina Castilla‐Ramirez, Carmen Nieves Hernandez‐Leon, Cleofe Romagosa, Maria Angeles Vaz Salgado, Javier Lavernia, Silvia Bagué, Empar Mayodormo‐Aranda, Luis Vicioso, Jose Emilio Hernández Barceló, Jordi Rubio‐Casadevall, Ana de Juan, Maria Concepcion Fiaño‐Valverde, Nadia Hindi, Maria Lopez‐Alvarez, Serena Lacerenza, Joaquin Dopazo, Antonio Gutierrez, Rosa Alvarez, Claudia Valverde, Javier Martinez‐Trufero, Javier Martín‐Broto

**Affiliations:** ^1^ Institute of Biomedicine of Seville (IBIS, HUVR, CSIC, Universidad de Sevilla) Spain; ^2^ Clinical Bioinformatics Area Fundación Progreso y Salud (FPS) CDCA Hospital Virgen del Rocio Seville Spain; ^3^ Bioinformatics in Rare Diseases (BiER) Centro de Investigación Biomédica en Red de Enfermedades Raras (CIBERER) FPS Hospital Virgen del Rocio Seville Spain; ^4^ Pathology Department Miguel Servet University Hospital Zaragoza Spain; ^5^ Pathology Department Gregorio Marañon University Hospital Madrid Spain; ^6^ Pathological Anatomy Service Complejo Asistencial Universitario de León Spain; ^7^ Pathology Department Son Espases University Hospital Mallorca Spain; ^8^ Pathology Department Hospital Clinico San Carlos Madrid Spain; ^9^ Pathology Department Ciudad Real General Hospital Spain; ^10^ Pathology Department University Hospital Virgen del Rocio Seville Spain; ^11^ Pathology Department Canarias University Hospital Santa Cruz de Tenerife Spain; ^12^ Pathology Department Vall d'Hebron University Hospital Barcelona Spain; ^13^ Medical Oncology Department Ramon y Cajal University Hospital Madrid Spain; ^14^ Medical Oncology Department Instituto Valenciano de Oncologia Valencia Spain; ^15^ Pathology Service Hospital de la Santa Creu i Sant Pau Barcelona Spain; ^16^ Pathology Department Hospital Universitari i Politècnic la Fe Valencia Spain; ^17^ Pathology Department Virgen de la Victoria University Hospital Malaga Spain; ^18^ Pathology Department Virgen de la Arrixaca University Hospital Murcia Spain; ^19^ Medical Oncology Department Hospital Josep Trueta Catalan Institute of Oncology Girona Spain; ^20^ Medical Oncology Department Marqués de Valdecilla University Hospital Santander Spain; ^21^ Department of Histopathology Álvaro Cunqueiro Hospital University Hospital Complex of Vigo Spain; ^22^ Medical Oncology Department University Hospital Fundación Jimenez Diaz Madrid Spain; ^23^ University Hospital General de Villalba Madrid Spain; ^24^ Instituto de Investigacion Sanitaria Fundacion Jimenez Diaz (IIS/FJD) Madrid Spain; ^25^ INB‐ELIXIR‐es FPS Hospital Virgen del Rocío Seville Spain; ^26^ Hematology Department Son Espases University Hospital Mallorca Spain; ^27^ Medical Oncology Department Gregorio Marañon University Hospital Madrid Spain; ^28^ Medical Oncology Department Vall d'Hebron University Hospital Barcelona Spain; ^29^ Medical Oncology Department Miguel Servet University Hospital Zaragoza Spain

**Keywords:** gene signature, predictive biomarkers, trabectedin

## Abstract

Predictive biomarkers of trabectedin represent an unmet need in advanced soft‐tissue sarcomas (STS). DNA damage repair (DDR) genes, involved in homologous recombination or nucleotide excision repair, had been previously described as biomarkers of trabectedin resistance or sensitivity, respectively. The majority of these studies only focused on specific factors (*ERCC1*, *ERCC5,* and *BRCA1*) and did not evaluate several other DDR‐related genes that could have a relevant role for trabectedin efficacy. In this retrospective translational study, 118 genes involved in DDR were evaluated to determine, by transcriptomics, a predictive gene signature of trabectedin efficacy. A six‐gene predictive signature of trabectedin efficacy was built in a series of 139 tumor samples from patients with advanced STS. Patients in the high‐risk gene signature group showed a significantly worse progression‐free survival compared with patients in the low‐risk group (2.1 vs 6.0 months, respectively). Differential gene expression analysis defined new potential predictive biomarkers of trabectedin sensitivity (*PARP3* and *CCNH)* or resistance (*DNAJB11* and *PARP1*). Our study identified a new gene signature that significantly predicts patients with higher probability to respond to treatment with trabectedin. Targeting some genes of this signature emerges as a potential strategy to enhance trabectedin efficacy.

## Graphical abstract

By using direct transcriptomics, we identified a new DNA damage repair gene‐associated signature with predictive value for trabectedin efficacy in soft‐tissue sarcoma (STS). Patients in the high‐risk gene signature group showed a significantly worse progression‐free survival compared to patients in the low‐risk group. These findings may help in identifying new therapeutic targets that could enhance the efficacy of trabectedin in STS.

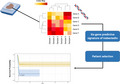

**Abbreviations**
AP: apurinic/apyrimidinic
AUC: area under the ROC curve
CR: complete response
DDR: DNA damage repair
DSB: double‐strand break
FC: fold change
FDR: false discovery rate
FFPE: formalin‐fixed paraffin‐embedded
GMI: growth modulation index
HR: homologous recombination
NER: nucleotide excision repair
NHEJ: non‐homologous end joining
OS: overall survival
PD: progression disease
PFS: progression‐free survival
PR: partial response
SD: stable disease
STS: soft‐tissue sarcoma
TMM: trimmed mean of M‐values
TPM: transcripts per million
TTP: time‐to‐progression

## 1 Introduction

Sarcomas are a heterogeneous group of rare malignant tumors with mesenchymal origin that can affect soft, bone, and visceral tissue. Sarcoma incidence is around 50–60 new cases per year for every million people, 20% being metastatic at diagnosis. Around 30–40% of localized sarcomas will eventually develop metastasis. In other words, a half of sarcoma population will suffer, sooner or later, metastatic spread. For locally advanced or metastatic soft‐tissue sarcoma (STS), the therapeutic options are scarce and the Response Evaluation Criteria in Solid Tumors (RECIST) responses to chemotherapy are infrequent, while the median of survival ranges between 18 and 22 months [1]. While doxorubicin is the backbone of first‐line therapy for advanced disease, second‐line treatments include pazopanib and chemotherapeutic agents such as trabectedin, eribulin, and gemcitabine combinations. The median progression‐free survival (PFS) in first‐line therapy for STS ranges between 4.5 and 6.0 months, whereas the median PFS for most of the second‐line drugs is below 5 months. Around 50% of the patients obtain a clinical benefit from these drugs [2, 3].

Trabectedin is an antitumor drug approved for the treatment of adult patients with advanced STS after failure of anthracyclines and ifosfamide, or for whom are unsuited to receive these agents [4, 5]. This compound is a DNA‐binding agent that interferes with oncogenic gene transcription, which has been described as its main mechanism of action. Besides, the transcription‐coupled nucleotide excision repair (NER) DNA repair machinery, in charge of repairing bulky adducts in DNA, is tricked by the special conformation of trabectedin adducts, ultimately inducing double‐strand breaks (DSBs) in the DNA. This process generates cell cycle perturbations, with a delayed S phase progression and accumulation of cells in G2 phase [6]. Additionally, trabectedin seems to affect also transcription of cytokines and growth factors in tumor‐associated macrophages and circulating monocytes [7, 8], and it sensitizes cancer cells to FAS receptor programmed cell death [9]. It has been also suggested that trabectedin could be a good radiosensitizer, since it causes G2/M arrest, which is the most sensitive cell cycle phase to radiation [10]. The high efficacy of this combination was observed in phase I/II trials in patients with metastatic STS of diverse histologies and patients with localized myxoid liposarcoma [11, 12].

Trabectedin treatment triggers DNA damage repair (DDR). Therefore, DDR‐related factors might be potential predictive biomarkers for this drug. Few retrospective studies supported this observation and reported that overexpression of genes involved in NER machinery (e.g., *ERCC1* and *ERCC5*) behaved as predictive biomarkers for trabectedin efficacy, whereas the overexpression of homologous recombination (HR)‐associated factors (e.g., *BRCA1*) was associated with trabectedin resistance [13, 14, 15]. In line with this, the potential predictive value of *ERCC1* and *ERCC5* was also validated by our group, in a cohort of cases prospectively collected from the randomized phase II trial that compared trabectedin plus doxorubicin versus doxorubicin alone as first line of advanced STS [16]. Nevertheless, the majority of these studies only focused on specific DDR‐related proteins/genes, such as *ERCC1*, *ERCC5,* and *BRCA1,* and did not analyze several other DDR‐related genes that could also have predictive relevance for trabectedin efficacy.

Accordingly, the aim of this retrospective translational study was to evaluate the expression of 118 genes involved in DDR, as potential predictive biomarkers of trabectedin activity and to determine whether any of these genes or a gene signature could predict trabectedin efficacy, in a more robust way than the already described expression of certain limited genes (i.e., *ERCC1*, *ERCC5,* and *BRCA1*). New biomarkers or novel insights on the mechanisms underlying drug activity may allow the development of new therapeutic options for STS and a better selection of patients for trabectedin treatment.

## 2 Methods

### 2.1 Patients and samples

A series of 140 formalin‐fixed paraffin‐embedded (FFPE) tumor samples, from STS patients treated with trabectedin in real‐life setting at any line of advanced disease, was selected from a Spanish Group for Research on Sarcomas (GEIS) registry and was the basis of this translational research. All the samples were collected with the informed consent signed from the patients, according to national regulations, and the study protocol was approved by the Ethics Committees and the institutional review board of each participant center. Study procedures were performed in accordance with the Declaration of Helsinki.

### 2.2 Oncology Biomarker Panel

The HTG EdgeSeq Oncology Biomarker Panel (OBP) direct‐transcriptomic assay quantitatively measures the expression of 2549 human RNA transcripts (https://www.htgmolecular.com/assays/obp) associated with tumor biology. Among these RNA transcripts, 118 are associated with DDR mechanisms and only these genes were considered for the purpose of this study. For direct transcriptomics, all the samples used in the assay had to have at least 70% of tumor area; samples with less than 70% tumor or with more than 20% of necrotic tissue underwent macro‐dissection. All the samples and controls were quantified in triplicate, and no template control was included in each run.

The RNA‐Seq libraries were synthesized through HTG EdgeSeq chemistry. In summary, this method consists of a first step that lysed and permeabilized the sample, which allows the release of the RNA. Then, nuclease protection probes were added to the lysed samples to hybridize with the exposed mRNA and target‐captured the transcripts. The S1 nuclease was added to the mix, allowing the production of a stoichiometric amount of target mRNA/nuclease protection probes duplexes. The reaction was blocked by denaturing S1 enzyme by heat. In order to reduce potential biases in the run, the samples were randomized before the inclusion in the HTG EdgeSeq system.

PCRs were set up using each hybridized sample as template and specially designed tags that shared common sequences complementary to 5′‐end and 3′‐sequences of the probes, as well as with common adaptors that are required for cluster generation on an Illumina sequencing platform. Additionally, each tag contains a unique barcode useful for sample identification and multiplexing. Agencourt AMPure XP (Beckman Coulter, Beverly, MA, USA) was used to clean the PCR amplification product.

Moreover, the libraries were quantified by quantitative PCR, using the KAPA Library Quantification (Roche, Basel, Switzerland) kit, according to the manufacturer's instructions. All samples and controls were quantified in triplicate, and no template control was included in every run. Library denaturation was performed by adding first 2 N NaOH to the library, followed by the addition of 2 N HCl. The PhiX was spiked in at a 5% (concentration of 12.5 pm).

One demultiplexed FASTQ file per sample was retrieved from the sequencer for data processing. The HTG EdgeSeq host software performed the alignment of the FASTQ files with the probe list, the results were parsed, and the output was obtained as a read counts matrix. The HTG EdgeSeq was run in the VERIP service laboratory of HTG in Tucson (HTG Molecular Diagnostics, Tucson, AZ, USA).

### 2.3 Data filtering and normalization

Negative control probes were used as quality control to evaluate baseline performance, as previously described by Chadly *et al*. [17]. Briefly, the mean of negative probes for each sample was calculated, and the difference between negative control average and the mean of all negative control probes was obtained (Δ^mean^). Those samples with a Δ^mean^ outside the bounds of ±2 standard deviation were excluded from the study. Of 140 sample initially included in the study, seven were removed from the analysis. Data normalization was performed by applying trimmed mean of *M*‐values method (TMM) using edger package from r/bioconductor, adjusting for the total reads within a sample [18].

### 2.4 Differential gene expression analysis

For differential gene expression analysis, samples were grouped according to PFS, growth modulation index (GMI), or clinical benefit/response of trabectedin. PFS was measured from the date of initial treatment with trabectedin (PFS) to the event of progression or death, whichever occurred first. Clinical benefit was defined as the percentage of patients achieving complete response (CR) or partial response (PR) and/or stable disease (SD). Response was evaluated according to RECIST v.1.1. GMI was expressed as a ratio of intrapatient successive time‐to‐progression (TTP): GMI = TTP under trabectedin/TTP for the treatment prior to trabectedin.

For PFS analysis, the low‐risk group (better prognosis) was made up of the cases with a PFS value higher than the median (3.2 months) and the high‐risk group (worse prognosis) comprised of the samples with PFS lower than the median. For GMI analysis, the low‐risk group comprised of samples with a GMI ≥ 1.33 and the high‐risk group the cases with a GMI < 1.33. For RECIST response or clinical benefit analysis, samples were grouped as both: cases with response versus cases with stable or progressive disease or cases with response or stable disease versus cases with progression disease (PD).

The differential gene expression between groups was evaluated by a negative binomial generalized log‐linear model, using the edger package and implementing the procedure proposed by Robinson and Smyth [19]. The correction for multiple comparisons of Benjamini and Hochberg was applied, and a *P*‐value threshold of 0.05 was set [20]. Fold change (FC) values were obtained, along with *P*‐values and adjusted *P*‐values (false discovery rate; FDR) for the 118 genes evaluated. The batch effect was taken into account by adding batch information to the model as a covariable. For data visualization and later analyses, normalized log‐TMM values were obtained and the variability due to batch effect was removed using the removeBatchEffect method implemented in limma r package.

All analyses were performed with r/bioconductor (3.10) running on r version 3.6.0, (https://www.r‐project.org/).

### 2.5 Statistical analysis

The variables that followed binomial distributions (e.g., patient demographics) were expressed as frequencies and percentages. Categorical variables were expressed as absolute and relative frequencies or as continuous variables as median, range (minimum–maximum). Gene expression levels were indicated as mean ± standard deviation. The comparisons between quantitative and qualitative variables were performed through *U* of Mann–Whitney nonparametric test. FDR was used to adjust for multiple comparisons. PFS and overall survival (OS) from trabectedin initiation were estimated according to the Kaplan–Meier method. The log‐rank test was used to determine the associations between the variables of interest (i.e., gene expression and clinical outcomes). The *P*‐values reported were 2‐sided, and the statistical significance was defined at *P* ≤ 0.05. Statistical analysis was performed with spss 22.0 software (IBM, Armonk, NY, USA).

### 2.6 Construction of a predictive signature based on gene expression

Raw counts were normalized to transcripts per million (TPM), as it is a more appropriate unit to compare expression across samples than normalized counts from other methods such as deseq2 and edger. Of 140 cases included, only one patient with PFS = 0 was removed from the predictive gene signature analysis.

Univariate Cox regression analysis using the survival package was performed to find individual genes whose expression was significantly associated (*P* < 0.05) with PFS. Additionally, patients were divided, for each gene, into two groups, using an optimal cutoff from the survminer package which maximized the separation of Kaplan–Meier curves of every group, and only those genes with significantly different curves (*P* < 0.05) were further retained.

The remaining genes were used as input to build a gene expression signature using a multivariate Cox regression applying a Lasso penalty to minimize the risk of overfitting with the glmnet package. The penalty parameter was calculated using a fourfold cross‐validation (at the minimum partial likelihood deviance) of the complete cohort, randomly dividing 75% and 25% of patients into training and testing sets, respectively, 10 000 times.

Risk scores were calculated by multiplying the expression of every gene with its corresponding Cox regression coefficient, for each patient:

Riskscore=∑CoxregressioncoefficientofGenei×expressionofgeneGenei.

All analyses were performed in r (version 4.0.3).

## 3 Results

### 3.1 Patient demographics

A subset of 140 STS patients treated with trabectedin at line of advance disease with FFPE tumor sample available for RNA expression analysis was included in this translational study. The median age was 51 (17–79) years with 54% (*n* = 75) being female. Among the 140 STS included, 81% (*n* = 114) were localized at diagnosis, with 66% (*n* = 93) and 34% (*n* = 47) having a somatic or visceral primary tumor location, respectively. Of 140 cases, 59% (*n* = 83) were grade 3, 24% (*n* = 33) were grade 2, and 13% (*n* = 18) were grade 1. The most frequent subtypes were leiomyosarcoma (*n* = 44; 31%), liposarcoma (*n* = 32; 23%), synovial sarcoma (*n* = 14; 10%), and undifferentiated pleomorphic sarcoma (*n* = 14; 10%), while 26% (*n* = 36) were diagnosed with other sarcoma subtypes. Patient demographics are depicted in Table 1.

**Table 1:** Patient demographics.

	*N* (%)
Gender	
Male	65 (46)
Female	75 (54)
Stage at diagnosis	
Localized	114 (81)
Metastatic	26 (19)
Sarcoma subtype	
Leiomyosarcoma	44 (31)
Liposarcoma	32 (23)
Synovial sarcoma	14 (10)
Undifferentiated pleomorphic sarcoma	14 (10)
Other	36 (26)
Grade	
1	18 (13)
2	33 (24)
3	83 (59)
Not available	6 (4)
Location	
Somatic	93 (66)
Visceral	47 (34)
Median follow‐up from diagnostic (months)	45
Median follow‐up from trabectedin line (months)	12
Median age, years (range)	51 (17–79)

### 3.2 Trabectedin outcome and univariate analysis

With a median follow‐up of 45 months, the median PFS of trabectedin line was 3.5 months, and the median of OS measured from the trabectedin initiation was 12 months. There were 124 out of 140 patients evaluable for response by RECIST with the following distribution: 6 (4.8%) CR, 14 (11.3%) PR, 42 (33.9%) SD, and 62 (50.0%) PD as best response. The GMI was ≥ 1.33 in 40 (29%) cases, < 1.33 in 97 (69%) patients, whereas in 3 (2%) cases, this value was not calculable.

In the univariate analysis, grade 3 tumors were associated with a worse PFS [3.0 months (95% CI 2.2–3.8) vs 6.5 months (95% CI 2.0–11.0); *P* = 0.003] and worse OS [10.2 months (95% CI 5.7–14.7) vs 17.5 months (95% CI 13.9–21.1); *P* = 0.041], and diagnosis of L‐sarcoma subtypes was correlated with a better PFS [6.1 months (95% CI 3.6–8.5) vs 3.0 months (95% CI 2.0–4.0); *P* = 0.001] and better OS [18.2 months (95% CI 13.2–23.1) vs 7.2 months (95% CI 3.7–10.8); *P* = 0.001]. Somatic location was associated with a better PFS in our series [5.6 months (95% CI 3.2–7.9) vs 2.7 months (95% CI 2.1–3.2); *P* = 0.006]. Patients receiving trabectedin in first or second line had better PFS [6.3 months (95% CI 1.0–11.5) vs 3.2 months (95% CI: 2.4–4.0); *P* = 0.001] (Table 2). Visceral location [HR = 1.9 (95% CI 1.3–2.8), *P* = 0.002] and non‐L‐sarcoma [HR = 1.9 (95% CI 1.3–2.9), *P* = 0.001] were independent factors associated with a worse PFS of trabectedin in the multivariate analysis.

**Table 2:** Univariate analysis of clinical factors.

Factor	PFS (95% CI)	*P*	OS (95% CI)	*P*
Sex		0.276		0.613
Female	3.2 (2.5–4.0)		13.1 (8.0–18.2)	
Male	4.4 (2.5–6.3)		13.1 (4.8–21.5)	
Age		0.178		0.674
< 51	5.1 (2.2–8.0)		12.5 (5.8–19.2)	
> 51	3.0 (2.1–3.9)		13.1 (6.2–20.1)	
Subtype		0.001		0.001
L‐sarcoma	6.1 (3.6–8.5)		18.2 (13.2–23.1)	
Non‐L‐sarcoma	3.0 (2.0–4.0)		7.2 (3.7–10.8)	
Grade		0.003		0.041
1 and 2	6.5 (2.0–11.0)		17.5 (13.9–21.1)	
3	3.0 (2.2–3.8)		10.2 (5.7–14.7)	
Location		0.006		0.051
Somatic	5.6 (3.2–7.9)		17.0 (10.2–23.9)	
Visceral	2.7 (2.1–3.2)		11.3 (7.6–15.0)	
Stage at diagnosis		0.057		0.523
Localized	4.4 (2.1–6.7)		13.8 (8.6–19.0)	
Metastatic	3.0 (2.2–3.8)		5.4 (0.0–16.7)	
Trabectedin line		0.001		0.218
1/2	6.3 (1.0–11.5)		17.0 (9.5–24.6)	
> 2	3.2 (2.4–4.0)		12.0 (5.8–18.1)	

### 3.3 Expression of DNA damage repair genes in STS

The expression of DDR‐related genes was heterogeneous, being *DNAJB8* (log2 2.80 ± 1.45) and *APEX1* (log2 9.97 ± 0.66) the most underexpressed and overexpressed genes, respectively, among the transcripts analyzed (Table S1).

Considering the expression of DDR‐related transcripts in relation to the clinical variables grouped as: L‐sarcomas vs non‐L‐sarcomas and grade 3 vs grade 1–2, the outcome was as follows: of 118 genes analyzed, only two genes were significantly overexpressed (i.e., *BRIP1*, *DNAJB5*) in L‐sarcomas, compared with non‐L‐sarcomas, whereas 16 genes were significantly underexpressed (i.e., *DDB2*, *DNAJA1*, *DNAJA2*, *DNAJB2*, *DNAJC10*, *DNAJC14*, *DNAJC16*, *ERCC3*, *ERCC6*, *MMS19*, *MSH2*, *PMS2*, *SMUG1*, *TDG,* and *XRCC5*) in L‐sarcomas, compared with other STS subtypes. Grade 3 tumors showed a significant overexpression, compared with grade 1–2 cases, of *APEX2*, *BRIP1*, *DNAJC1*, *DNAJC11*, *EXO1*, *FEN1*, *MSH2*, *MSH6*, *NEIL2*, *NEIL3*, *PRKDC*, *RAD21*, *RAD51*, *RAD54L*, *RPA3*, *TREX1,* and *XRCC3*, whereas *ATM* and *XPC* were overexpressed in this latter group, compared with grade 3 tumors (Table S2).

### 3.4 Differential gene expression and univariate analysis

Differential gene expression analysis was carried out in 133 cases after data normalization.

Twenty genes were significantly and differently expressed, by multiple comparisons (FDR < 0.05), for the PFS of trabectedin (Table 3 and Fig. S1). High expression of *PARP3* (logFC = 1.738; FDR < 0.001), *POLL* (logFC = 1.767; FDR = 0.002), *PMS1* (logFC = 0.764; FDR = 0.003), *RAD52* (logFC = 0.875; FDR = 0.003), *ATM* (logFC = 0.444; FDR = 0.014), *NEIL1* (logFC = 0.773; FDR = 0.021), *TOP3B* (logFC = 1.374; FDR = 0.030), *CCNH* (logFC = 0.620; FDR = 0.030), *XRCC5* (logFC = 0.862; FDR = 0.030), *DDB2* (logFC = 0.314; FDR = 0.031), *MUTYH* (logFC = 0.369; FDR = 0.034), *DNAJB14* (logFC = 0.420; FDR = 0.035), and *XPC* (logFC = 0.512; FDR = 0.037) was associated with longer PFS, whereas the overexpression of *DNAJB11* (logFC = −0.378; FDR = 0.003), *PARP1* (logFC = −0.442; FDR = 0.007), *TDG* (logFC = −0.312; FDR = 0.030), *TOP3A* (logFC = −0.451; FDR = 0.030), *PRKDC* (logFC = −0.392; FDR = 0.030), *DNAJC11* (logFC = −0.309; FDR = 0.030), and *RAD23B* (logFC = −0.311; FDR = 0.035) were associated with worse PFS.

**Table 3:**
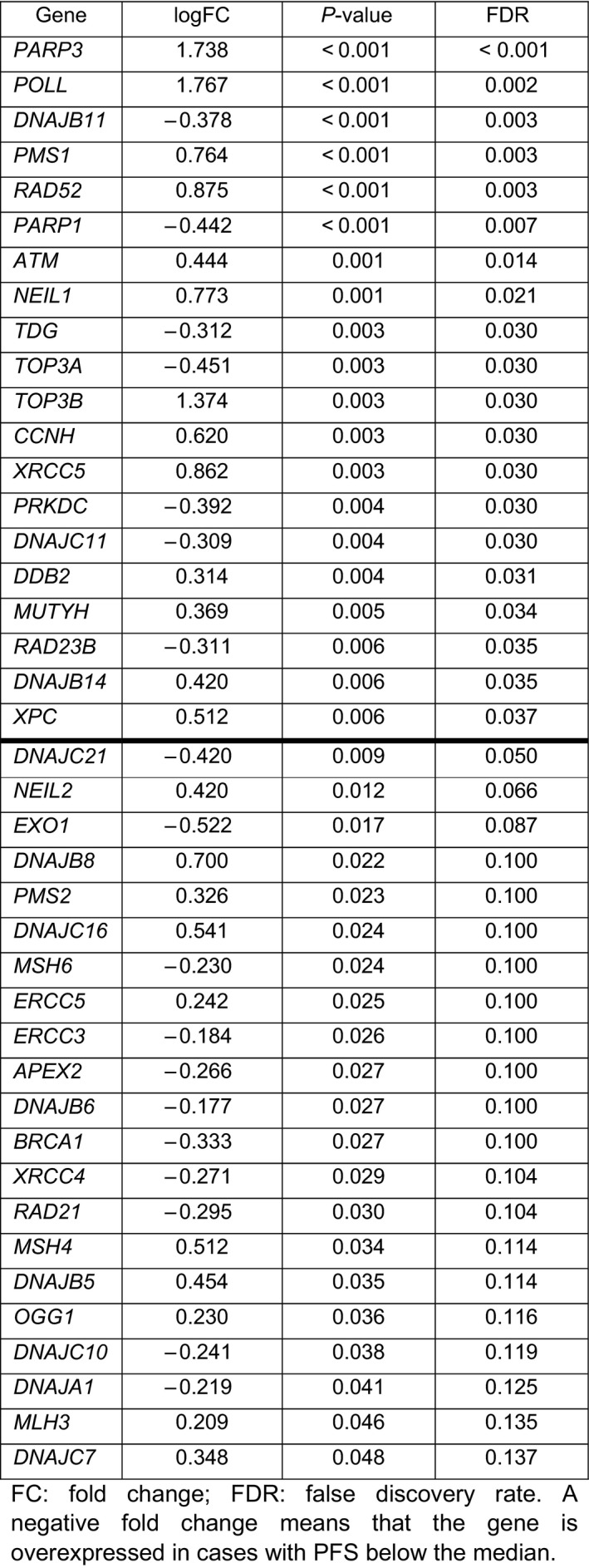
Differential gene expression attending to median PFS (3.2 months). A negative fold change means that the gene is overexpressed in cases with PFS below the median.

**Fig. 1:**
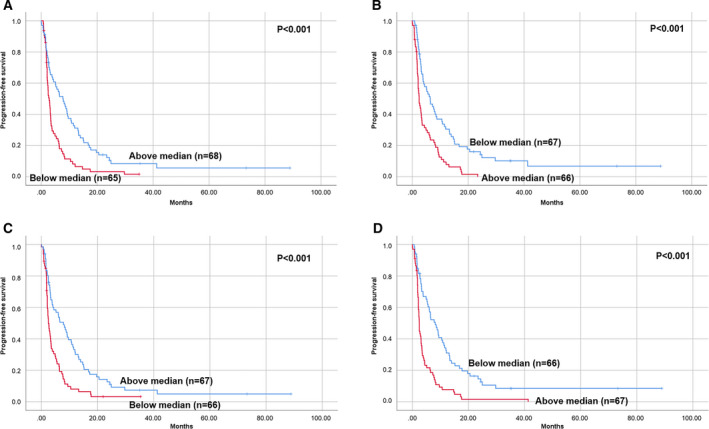
Univariate analysis. (A) PFS according to *PARP3* gene expression; (B) PFS according to *PARP1* gene expression; (C) PFS according to *CCNH* gene expression, and (D) PFS according to *DNAJB11* gene expression. Groups were defined according to the median of gene expression; above and below median. Log‐rank test statistical significance was defined at *P* ≤ 0.05.

The univariate analysis revealed that *PARP3* and *CCNH* overexpression was markedly related to a longer PFS of trabectedin: 7.7 months (95% CI 4.6–10.9) vs 2.8 months (95% CI 2.1–3.6), *P* < 0.001 and 7.9 months (95% CI 4.8–11.0) vs 2.5 months (95% CI 1.9–3.0), *P* < 0.001, respectively. On the other extreme, *DNAJB11* and *PARP1* overexpression was notably related to a shorter PFS of trabectedin: 2.5 months (95% CI 2.3–3.7) vs 8.2 months (95% CI 5.2–11.1), *P* < 0.001 and 2.5 months (95% CI 1.8–3.1) vs 6.4 months (95% CI 4.0–8.8), *P* < 0.001, respectively (Fig. 1 and Table S3). The remaining genes with statistical significance for the PFS of trabectedin, as well as their impact in OS from trabectedin initiation, are represented in Table S3.

The overexpression of *BRCA1* gene was significantly associated with worse PFS of trabectedin [2.5 months (95% CI 1.6–3.3) vs 6.1 months (95% CI 2.5–9.7), *P* = 0.001]; however, neither the expression of *ERCC1* [5.2 months (95% CI 2.5–8.0) vs 3.0 months (95% CI 2.2–3.9), *P* = 0.084] nor of *ERCC5* [3.2 months (95% CI 2.3–4.1) vs 4.4 months (95% CI 1.4–7.4), *P* = 0.182] showed a significant association with PFS of trabectedin (Table S3).

Considering GMI values, grouped as GMI ≥ 1.33 vs GMI < 1.33, only six genes were differentially expressed with statistical significance between both groups (FDR < 0.05). Patients with a GMI ≥ 1.33 showed significantly higher expression of *DNAJC16* (logFC = 1.163; FDR = 0.001), *XPC* (logFC = 0.799; FDR = 0.004), *DNAJB14* (logFC = 0.611; FDR = 0.006), *ATM* (logFC = 0.523; FDR = 0.008), *DNAJC7* (logFC = 0.684; FDR = 0.008), and *NEIL2* (logFC = 0.564; FDR = 0.039), compared with those with a GMI < 1.33 (Table 4).

**Table 4:**
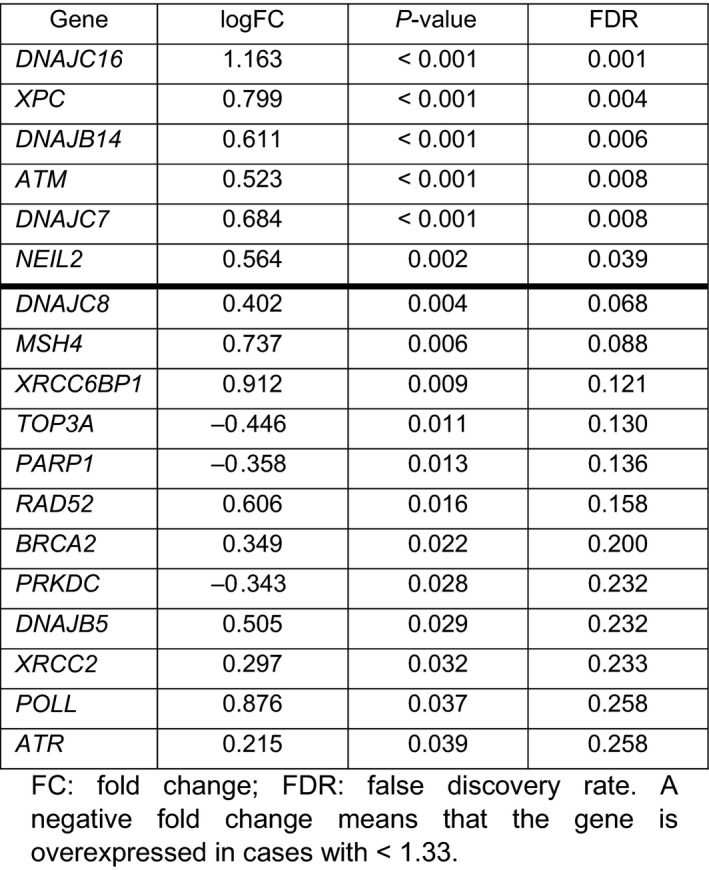
Differential gene expression attending to growth modulation index. A negative fold change means that the gene is overexpressed in cases with < 1.33.

Differential gene expression reached statistical significance in 20 genes when it was considered obtaining or not clinical benefit (CR+PR+SD vs PD) (Table 5). High expression of *PARP3* (logFC = 1.538; FDR = 0.007), *POLL* (logFC = 1.876; FDR = 0.007), *RAD52* (logFC = 0.849; FDR = 0.009), *TOP3B* (logFC = 1.818; FDR = 0.009), *XRCC5* (logFC = 1.073; FDR = 0.009), *DNAJB14* (logFC = 0.463; FDR = 0.032), *XPC* (logFC = 0.559; FDR = 0.036), *ATM* (logFC = 0.383; FDR = 0.036), *MSH5* (logFC = 0.843; FDR = 0.043), and *NEIL2* (logFC = 0.467; FDR = 0.043) were associated with clinical benefit of trabectedin, whereas overexpression of *RAD21* (logFC = −0.522; FDR = 0.007), *MSH6* (logFC = −0.369: FDR = 0.009), *DNAJB11* (logFC = −0.336; FDR = 0.011), *TDG* (logFC = −0.332; FDR = 0.024), *XRCC4* (logFC = −0.394; FDR = 0.024), *PARP1* (logFC = −0.373; FDR = 0.036), *ERCC3* (logFC = −0.242; FDR = 0.036), *PRKDC* (logFC = −0.380; FDR = 0.043), *RAD23B* (logFC = −0.315; FDR = 0.043), and *TOP3A* (logFC = −0.423; FDR = 0.043) were associated with progressive disease after trabectedin. By contrast, no different gene expression was significantly detected, by FDR, when the grouping was responders (CR+PR) vs non‐responders patients (Table S4).

**Table 5:**
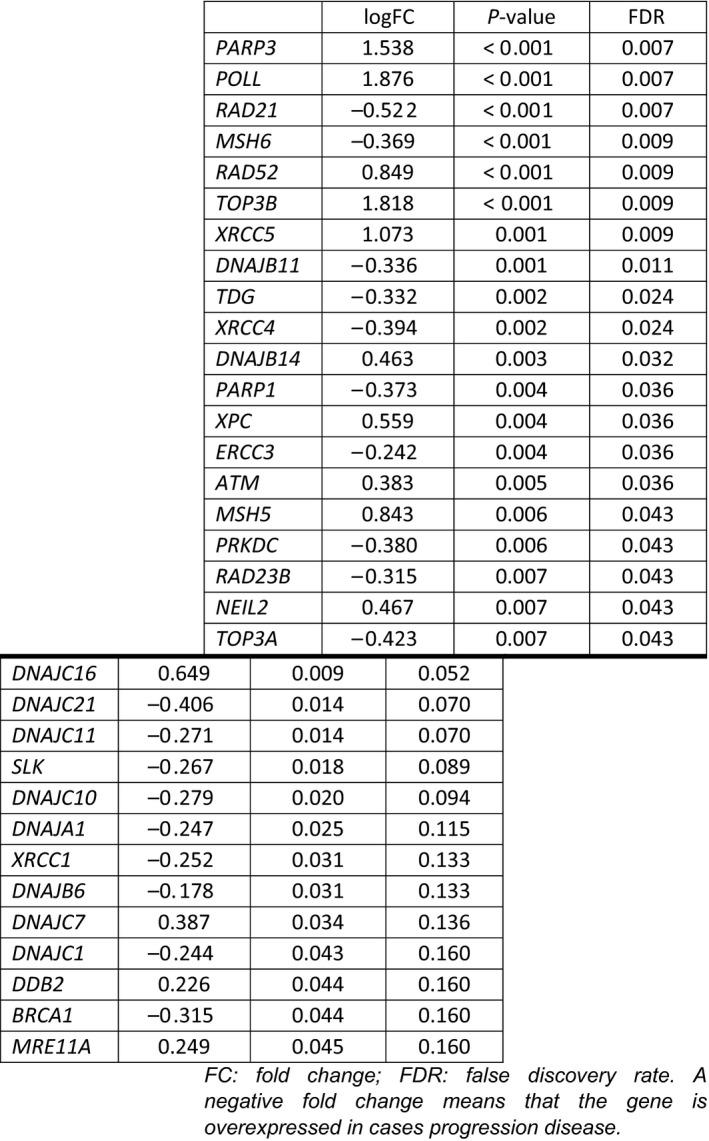
Differential gene expression according to clinical benefit. A negative fold change means that the gene is overexpressed in cases progression disease.

Regarding the different analyses performed, only three genes were significantly associated with a longer PFS (Table 3), GMI ≥ 1.33 (Table 4), and clinical benefit of trabectedin (Table 5): *ATM*, *DNAJB14,* and *XPC* (Fig. S2). Further, 14 significantly expressed genes were common for PFS and clinical benefit endpoints: *ATM*, *DNAJB11*, *DNAJB14*, *PARP1*, *PARP3*, *POLL*, *PRKDC*, *RAD23B*, *RAD52*, *TDG*, *TOP3A*, *TOP3B*, *XPC,* and *XRCC5* (Fig. S2).

Differential gene expression was also determined according to clinical variables with an independent value for PFS in the multivariate analysis: Table S5 (L‐sarcomas vs other subtypes) and Table S6 (visceral vs somatic location). *PARP3* and *PMS1*, which were genes associated with a better PFS of trabectedin, were overexpressed in L‐sarcomas and in tumors with a somatic location, whereas *PARP1*, a gene that correlated with worse PFS of trabectedin, was highly expressed in non‐L‐sarcomas and tumors with visceral location.

### 3.5 Predictive gene signature of trabectedin activity

Univariate Cox regression model on a cohort of 139 patients identified eight genes whose expression was significantly associated with PFS of trabectedin. Further selection of genes whose Kaplan–Meier curves displayed significant differences (patients were divided into two groups based on gene expression median value) yielded six final genes. Lasso‐penalized Cox regression analysis was performed to construct a predictive gene signature for trabectedin. The six genes identified were as follows: exonuclease 1 (*EXO1*), cyclin H (*CCNH*), xeroderma pigmentosum group A‐complementing protein (*XPA*), poly(ADP‐ribose) polymerase 1 (*PARP1*), *BRCA1,* and apurinic/apyrimidinic endodeoxyribonuclease 2 (*APEX2*).

$$\begin{array}{l} \begin{array}{cc} \text{Risk}\;\text{score} & =0.1866930\times {\text{Expression}}_{EXO1}+\left(‐0.1966279\right)\times \\ & {\text{Expression}}_{\mathrm{CCNH}}\\ & \;+\left(‐0.2043687\right)\times {\text{Expression}}_{\mathrm{XPA}}+0.2293783\times \\ & {\text{Expression}}_{PARP1}\\ & \;+0.0129766\times {\text{Expression}}_{BRCA1}+0.2278331\times \\ & {\text{Expression}}_{APEX2}. \end{array} \end{array}$$

**Fig. 2:**
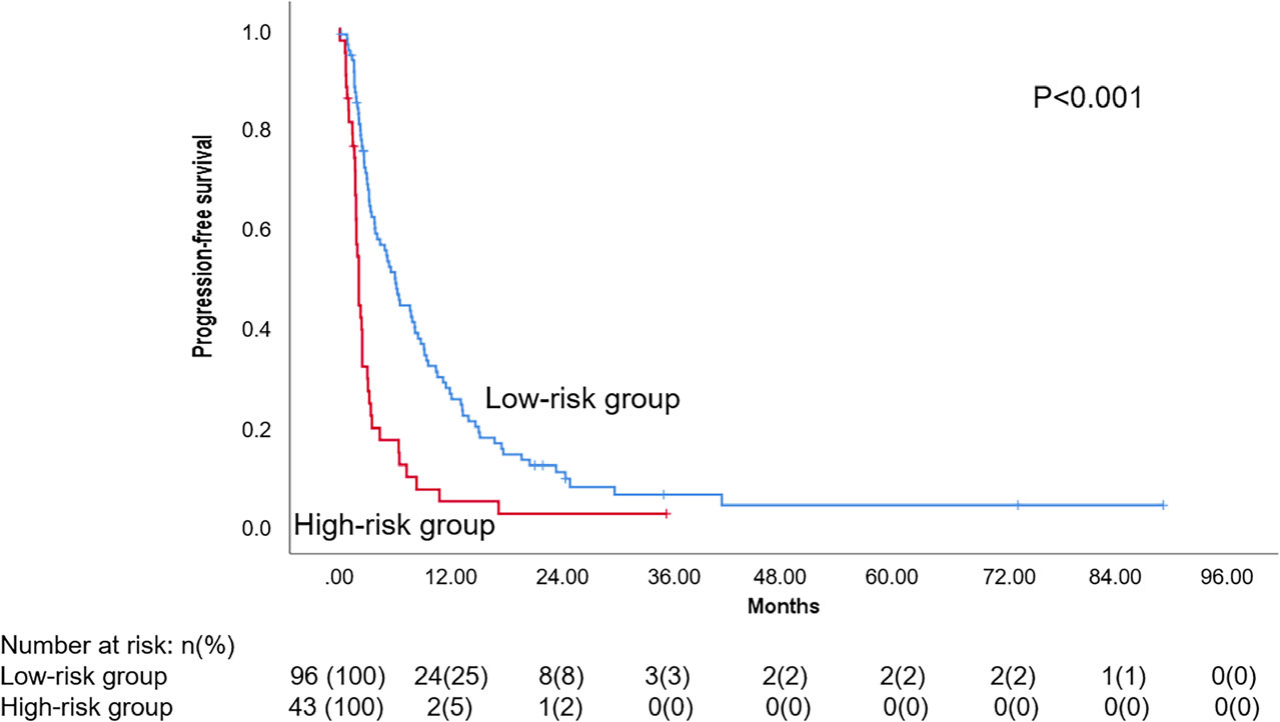
Risk groups Kaplan–Meier curve. Patients were grouped according to the risk scores cutoff value (2.146). Number at risk was represented as *n* (%). Log‐rank test statistical significance was defined at *P* ≤ 0.05.

The six‐gene‐based risk score was calculated for each patient, and the optimal cutoff for the risk score (2.146) was determined. An additional univariate Cox regression analysis was performed using risk scores, resulting in a significant association with PFS (Table S7). Time‐dependent ROC and Kaplan–Meier curves were used to assess the predictive value of the gene signature. The AUCs (area under the ROC curve) for 3‐, 12‐, and 24‐month PFS were 0.67, 0.65, and 0.65, respectively (Fig. S3). Patients in the high‐risk group (higher expression of *EXO1*, *PARP1*, *BRCA1,* and *APEX2* and lower expression of *CCNH* and *XPA*) showed a significantly worse PFS compared with the patients in the low‐risk group (lower expression of *EXO1*, *PARP1*, *BRCA1,* and *APEX2* and higher expression of *CCNH* and *XPA*), [2.1 months (95% CI 1.8–2.4) vs 6.0 months (95% CI 4.5–7.5), *P* < 0.001 (Fig. 2)]. PFS rate at 6 months and at 12 months was 16% and 5%, respectively, for the high‐risk group and 48% and 25%, respectively, for the low‐risk group. In the multivariate analysis, the high‐risk group was an independent factor associated with PFS [HR 2.1 (95% CI 1.4–3.2), *P* < 0.001]; visceral location and non‐L‐sarcomas maintained the independent value (Table S8).

In the patients treated with either doxorubicin or gemcitabine, the DDR‐related gene signature was also correlated with the PFS of these drugs. The six‐gene‐based signature failed to significantly predict gemcitabine or doxorubicin activity, in terms of PFS, in our series (Table S9).

## 4 Discussion

A six‐gene predictive signature of trabectedin efficacy was built based on the transcriptomic analysis of 118 DDR‐related genes and PFS, in a retrospective series of 139 advanced STS patients. Previously described predictive genes for trabectedin efficacy, as those related to NER machinery, such as *ERCC1* and *ERCC5,* did not show any relevant prognostic role in our series. Rather, other DDR‐related genes showed more consistent prediction of trabectedin activity, either in terms of PFS, GMI, and/or clinical benefit. For this study, we decided to focus only in the relevance of DDR‐related genes, since the DNA DSBs induced by trabectedin along with the involvement of NER machinery and HR repair genes were previously reported as a potential relevant mechanism of trabectedin activity in STS patients [13, 15, 21]. However, the majority of these studies were limited to few DDR‐related genes/proteins as potential predictive biomarkers of trabectedin, in consequence many other potential predictive factors from the family of DNA repair genes have been unexplored in this regard. Hence, we aimed to evaluate, in a broader way, the predictive role of a large number of DDR‐related genes using HTG EdgeSeq next‐generation sequencing (NGS)‐adapted molecular profiling. Subsequently, an established six‐gene signature was able to markedly separate two significant different populations (favorable or unfavorable signature) for trabectedin efficacy in terms of PFS.

Even when *BRCA1* (expression levels or haplotype status) had been previously associated with the clinical benefit of trabectedin [15, 21], its predictive role was weaker than the gene signature. Overexpression of *XPA* gene, another component of the signature and a NER‐associated gene, was in line with previous reports that showed a direct correlation between higher expression of NER machinery‐related genes and better trabectedin efficacy [22, 23]. Besides, and to the best of our knowledge, this is the first time that the expression of *EXO1*, *CCNH,* and *APEX2* had been associated with trabectedin efficacy. *EXO1* is an XPG family nuclease that participates in several DDR pathways, such as DNA mismatch repair, HR, and non‐homologous end joining (NHEJ) [22]. Of note, the expression of EXO1 was associated with resistance to cisplatin and doxorubicin via NHEJ in ovarian cancer, a tumor also sensitive to trabectedin. Nevertheless, the prognostic/predictive value of EXO1 in STS is still unknown [24, 25]. Cyclin H, coded by the *CCNH* gene, is a protein that forms a cyclin‐activating kinase‐subcomplex with CDK7 and MAT1. This subcomplex is part of the transcription factor II human (TFIIH) protein complex and it is involved in NER, by regulating cell cycling progression during DNA damage [26]. As expected in accordance with the role of cyclin H in NER pathway, a high expression of this gene was associated with trabectedin sensitivity in our signature. *APEX2* is a weak apurinic/apyrimidinic (AP) endonuclease in the DNA base excision repair pathway and functions in the context of DNA damage by oxidative and alkylating agents. APEX2 also exhibits strong activities of 3′–5′ exonuclease and 3′‐phosphodiesterase and that has been described to be necessary for the recovery of B‐cell lymphocyte progenitors after chemotherapy [27, 28]. Moreover, APEX2 has been identified as a synthetic lethal gene, in BRCA1‐ and BRCA2‐deficient cells [29]. APEX2 processes AP sites at replication forks, avoiding the blocking of fork progression and the induction of DSBs, which required BRCA1/2 for repair and DNA synthesis [29]. Of note, trabectedin has been described to cause replication impairment and genome instability, via RNA–DNA hybrid‐dependent DNA damage, which is important for drug activity [30]. Accordingly, it is possible that APEX2 may have a role in resolving replication impairment and genome instability induced by trabectedin, which may justify its association with lack of drug activity in our gene signature. This could also open the option to target APEX2 as a potential therapeutic strategy in combination with trabectedin if this gene is overexpressed. Remarkably, our six‐gene signature is in line with previous reports [13, 15, 16], wherein the high expression of NER genes (e.g., *XPA* and *CCNH*) and the low expression of HR genes (e.g., *EXO1* and *BRCA1*) would help to identify the STS patients that benefit from trabectedin treatment.

Furthermore, several DDR‐related genes were significantly associated with trabectedin activity, in terms of PFS, GMI, and/or clinical benefit. Among these genes, *PARP3* overexpression significantly associated with a better PFS and clinical benefit following trabectedin. *PARP3*, initially described in 1999 [31], is a poly(ADP ribosyl)transferase closely related with PARP1 and PARP2, and it is highly expressed in skeletal muscle [32]. *PARP3* is involved in transcriptional silencing and in the maintenance of genomic integrity [33], being part of the polycomb group complexes, which are relevant to maintain the silencing of specific critical genes related to development among others. More precisely, PARP3 was shown to interact with core proteins of the polycomb repressive complexes 2 and 3, such as the methyltransferases EZH2 and Suz12, which are relevant in the maintenance of repression through post‐translational methylation processes [33]. In turn, *PARP1* expression, a gene also included in our potential predictive molecular signature, was significantly associated with shorter PFS and lack of clinical benefit after trabectedin treatment. The distinct behavior or *PARP1* and *PARP3* genes in our study may indicate that these proteins may participate differently in some cellular mechanisms, which could affect the response to trabectedin. Both proteins are relevant for DNA genomic integrity [34]; however, it seems that PARP1 overexpression promotes cell cycle entry [35, 36, 37], whereas PARP3 overexpression interferes with G1/S cell cycle progression [38]. Of note, G1 seems to be the cell cycle phase in which cells are more sensitive to trabectedin [39]. Accordingly, the effect of the overexpression of each one of these proteins in cell cycle progression may justify, at least in part, the different results for both PARP1 and PARP3 in our study. Noteworthy, *PARP3* seems to be overexpressed in tumors that benefit from trabectedin treatment (i.e., L‐sarcomas and tumors with somatic location), while *PARP1* expression is higher in sarcomas with lower activity of trabectedin (non‐L‐sarcomas and tumors with visceral location). Therefore, the expression of these genes may be important biomarkers of trabectedin efficacy or resistance, in specific clinical contexts that depend on the histologic subtype or the location of the primary tumor. Besides, the association of *PARP1* high expression with worse trabectedin outcome supports the combination of PARP1 inhibitors with this chemotherapeutic agent. In line with these observations, the combination of olaparib with trabectedin has been tested in a phase 1b clinical trial in patients with advanced and non‐resectable bone and STS (ClinicalTrials.gov, number NCT02398058). Of 50 patients recruited, 7 had a PR, suggesting that this combination has antitumor activity [40]; however, patient selection based on PARP1 and/or PARP3 expression levels should be considered for further clinical trials with this combination.

In the study presented here, only *BRCA1* high expression was significantly associated with shorter PFS and OS, while *ERCC1* and *ERCC5* expression, which were previously associated with good prognosis to trabectedin [13, 15, 16], did not had a significant impact in survival. The different methodology used for gene expression analysis could justify the distinct results obtained in terms of survival observed for these two genes; however, it is important to mention that HTG quantitative nuclease protection assays for gene detections had been reported to have similar sensitivity as qPCR [41, 42]. Accordingly, our results may suggest that NER‐associated genes *ERCC1* and *ERCC5* are not robust and reliable biomarkers of trabectedin activity. Their predictive value should accordingly be considered with caution; however, a further study with a larger cohort of cases should validate our data.

Among the study limitations, it is important to mention that the tumor samples used for this retrospective analysis were collected at the diagnostic time, and gene expression levels could have been altered due to the several lines of previous treatment or during the natural progression of the disease. Moreover, another important limitation is related with its initial hypothesis; for this analysis, only genes related with DDR were selected and studied. This selection excluded, from the molecular signature, genes involved in other cellular processes, which may also predict trabectedin activity. Since we cannot exclude the relevance of other genes beyond DDR, further analysis, considering all the genes included in the OBP assay, is currently ongoing. The absence of an independent validation cohort, or a control cohort treated with any other drug approved for advanced STS, is other limitation of this study. The validation of the predictive value of the gene‐based signature in a larger series of cases could also help improve the performance of ROC curves.

## 5 Conclusions

In summary, this translational study identified a new molecular signature that could significantly predict patients with higher probability for trabectedin efficacy. This signature was based on wide transcriptomic analysis of DDR‐related genes and provides new insights for potential targets that could enhance the trabectedin efficacy. Validation of this signature is in process with an independent series of advanced STS treated with trabectedin. Finally, the potential value of specific biomarkers (e.g., PARP3) is currently being tested in the preclinical setting, in order to describe the mechanisms in which they participate under drug treatment.

## Conflict of interest

DSM reports institutional research grants from PharmaMar, Eisai, Immix BioPharma, and Novartis outside the submitted work and travel support from PharmaMar, Eisai, Celgene, Bayer, and Pfizer. NH declares Honoraria from PharmaMar and Lilly; Travel Grants from PharmaMar; Research Grants from PharmaMar, Eisai, Immix BioPharma, and Novartis outside the submitted work. Research funding for her Institution (clinical studies) from PharmaMar, Eli Lilly and Company, AROG, Bayer, Eisai, Lixte, Karyopharm, Deciphera, GSK, Novartis, Blueprint, Nektar, Forma, Amgen, Adaptimmune, and Daichii‐Sankyo. JM‐B: Honoraria from PharmaMar, Lilly, Bayer, and Eisai; travel grants from PharmaMar; research grants from PharmaMar, Eisai, Immix BioPharma, and Novartis outside the submitted work. Research funding for his institution (for clinical studies) from PharmaMar, Eli Lilly and Company, AROG, Bayer, Eisai, Lixte, Karyopharm, Deciphera, GSK, Novartis, Blueprint, Nektar, Forma, Amgen, Adaptimmune, and Daichii‐Sankyo.

## Author contributions

DSM and JM‐B designed the experiments and analysis. All the authors participated in data acquisition. DSM, MP‐C, JACV, and JM‐B performed data analysis and interpretation. DSM, MP‐C, JACV, and JM‐B drafted the manuscript. All the authors reviewed the manuscript and approved its final version.

## Supporting information


**Fig. S1.** Volcano plot of genes with prognostic relevance in progression‐free survival (PFS) of trabectedin.

**Fig. S2.** Venn diagram comparing the unique and shared genes between progression‐free survival, growth modulation index and clinical benefit.

**Fig. S3.** Time‐dependent ROC curve to assess the predictive value of the gene signature.

**Table S1.** Expression values of DNA damage repair‐related genes.

**Table S2.** Gene significantly expressed between L‐sarcoma and non‐L‐sarcomas and between grade 3 tumors vs grade 1 + 2 tumors.

**Table S3.** Univariate analysis of DNA damage repair‐related genes with impact in progression‐free survival to trabectedin line.

**Table S4.** Differential gene expression according to objective response.

**Table S5.** Differential gene expression according to histologic subtype grouping.

**Table S6.** Differential gene expression according to tumor location.

**Table S7.** Univariate Cox regression analysis for the values of the gene signature (risk scores).

**Table S8.** Multivariate analysis.

**Table S9.** Correlation between risk groups and progression‐free survival of doxorubicin or gemcitabine.

## Data Availability

The data that support the findings of this study are available from the corresponding author (jmartin@mustbesevilla.org) upon reasonable request.
