# Supplementary figures and images for: A DNA damage repair gene‐associated signature predicts responses of patients with advanced soft‐tissue sarcoma to treatment with trabectedin
Source: Mol Oncol. 2021 Jun 30;15(12):3691–705. doi: 10.1002/1878-0261.12996 (PMC8637557; doi:10.1002/1878-0261.12996)

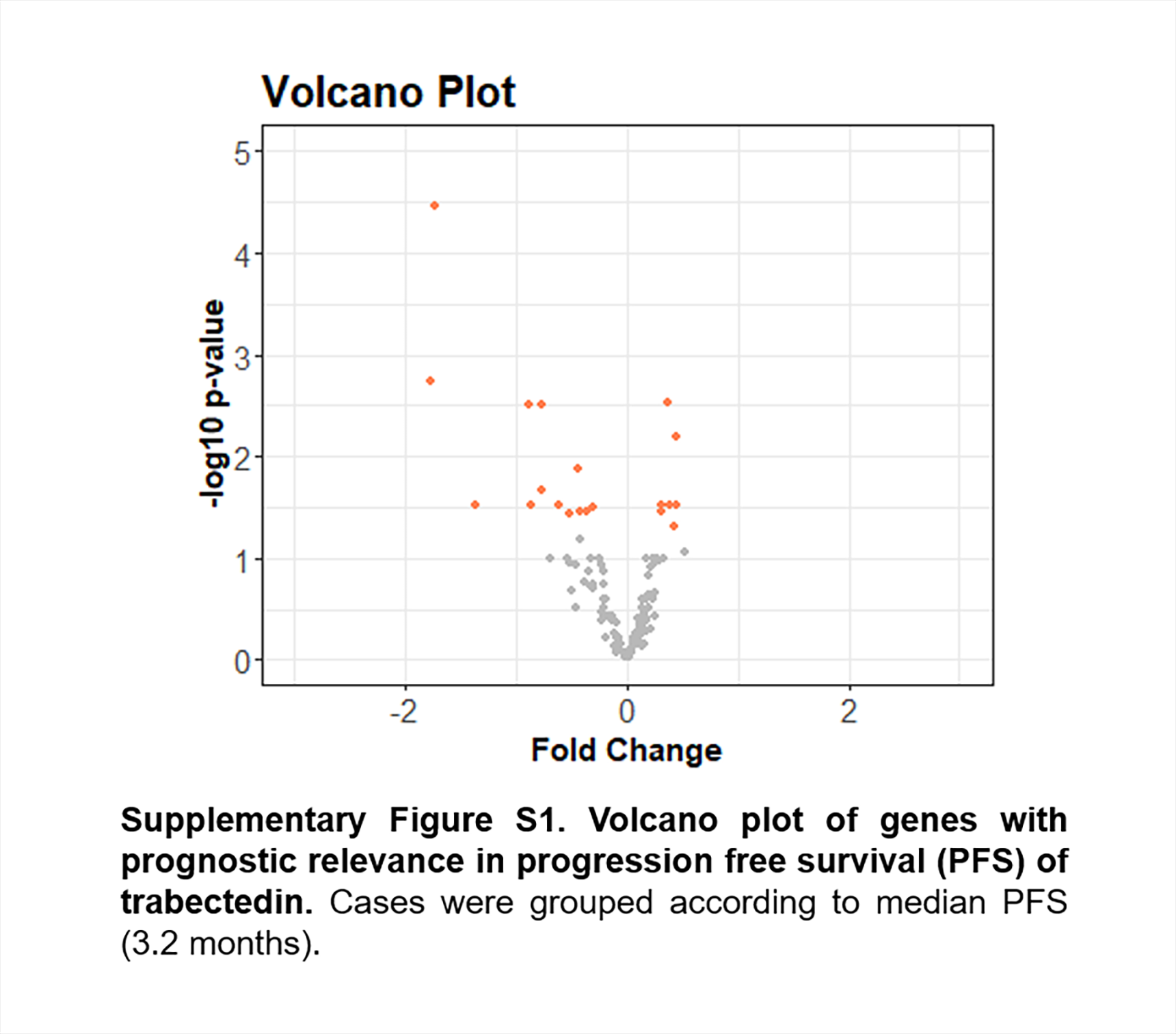

Supplement: Supplementary file 1 — Fig. S1. Volcano plot of genes with prognostic relevance in progression‐free survival (PFS) of trabectedin. [file MOL2-15-3691-s001.tif]

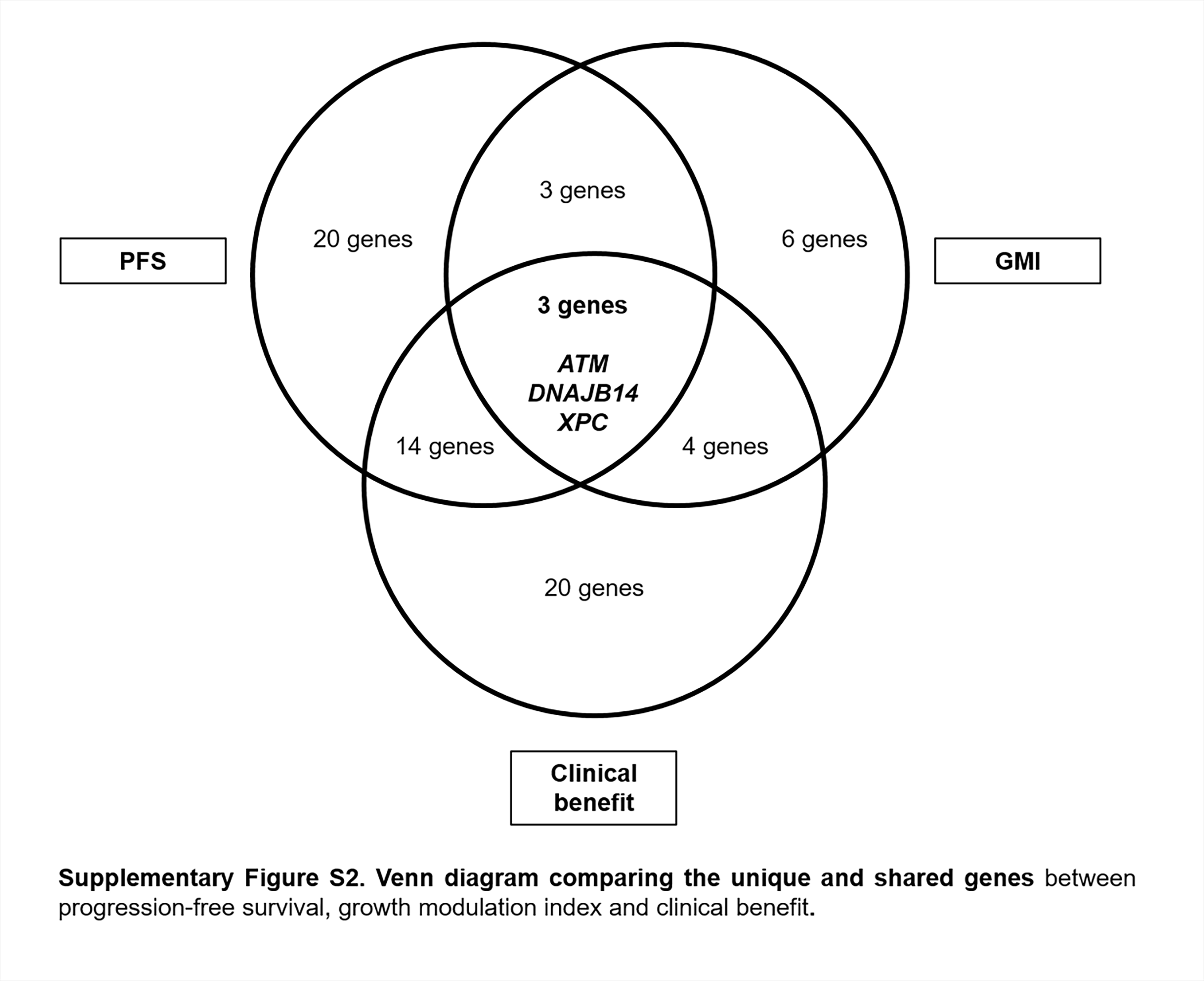

Supplement: Supplementary file 2 — Fig. S2. Venn diagram comparing the unique and shared genes between progression‐free survival, growth modulation index and clinical benefit. [file MOL2-15-3691-s005.tif]

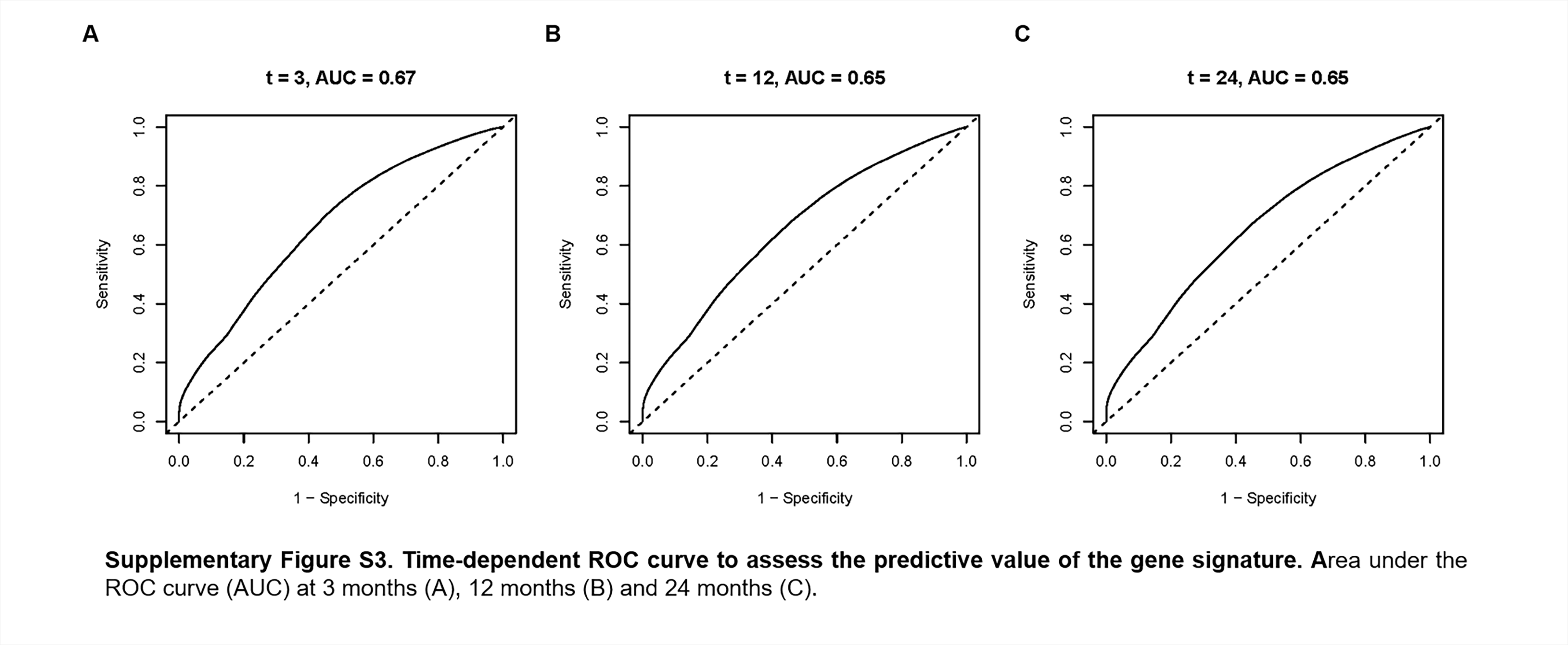

Supplement: Supplementary file 3 — Fig. S3. Time‐dependent ROC curve to assess the predictive value of the gene signature. [file MOL2-15-3691-s006.tif]
