# Supplementary material for: A DNA damage repair gene‐associated signature predicts responses of patients with advanced soft‐tissue sarcoma to treatment with trabectedin
Source: Mol Oncol. 2021 Jun 30;15(12):3691–705. doi: 10.1002/1878-0261.12996 (PMC8637557; doi:10.1002/1878-0261.12996)
Supplement: Supplementary file 4 — Table S1. Expression values of DNA damage repair‐related genes. [file MOL2-15-3691-s007.docx]

Supplementary Table S1. Expression values of DNA damage repair-related genes

| Gene | Mean expression | Standard Deviation |
| --- | --- | --- |
| *APEX1* | 9.97 | 0.66 |
| *APEX2* | 6.38 | 0.84 |
| *ATM* | 7.56 | 0.67 |
| *ATR* | 8.00 | 0.51 |
| *ATXN3* | 7.79 | 0.70 |
| *BRCA1* | 5.47 | 0.97 |
| *BRCA2* | 6.32 | 0.78 |
| *BRIP1* | 6.91 | 1.22 |
| *CCNH* | 3.60 | 1.23 |
| *CCNO* | 4.79 | 1.16 |
| *CDK7* | 6.93 | 0.48 |
| *DDB2* | 5.43 | 0.62 |
| *DMC1* | 3.44 | 1.63 |
| *DNAJA1* | 9.75 | 0.62 |
| *DNAJA2* | 5.79 | 0.92 |
| *DNAJA3* | 8.22 | 0.61 |
| *DNAJA4* | 7.01 | 0.88 |
| *DNAJB1* | 9.41 | 0.92 |
| *DNAJB11* | 8.29 | 0.56 |
| *DNAJB12* | 6.66 | 0.51 |
| *DNAJB13* | 3.97 | 1.52 |
| *DNAJB14* | 7.46 | 0.64 |
| *DNAJB2* | 7.49 | 0.71 |
| *DNAJB5* | 6.60 | 1.19 |
| *DNAJB6* | 9.25 | 0.45 |
| *DNAJB7* | 3.74 | 1.38 |
| *DNAJB8* | 2.80 | 1.45 |
| *DNAJB9* | 7.24 | 0.80 |
| *DNAJC1* | 8.02 | 0.66 |
| *DNAJC10* | 8.48 | 0.68 |
| *DNAJC11* | 7.35 | 0.61 |
| *DNAJC12* | 5.85 | 1.71 |
| *DNAJC13* | 8.08 | 0.57 |
| *DNAJC14* | 6.55 | 0.68 |
| *DNAJC15* | 6.89 | 1.15 |
| *DNAJC16* | 5.31 | 0.87 |
| *DNAJC17* | 7.65 | 0.46 |
| *DNAJC18* | 6.35 | 0.67 |
| *DNAJC21* | 6.09 | 0.91 |
| *DNAJC3* | 8.17 | 0.67 |
| *DNAJC4* | 7.30 | 0.67 |
| *DNAJC5* | 8.57 | 0.77 |
| *DNAJC5B* | 3.53 | 1.53 |
| *DNAJC5G* | 3.22 | 1.73 |
| *DNAJC6* | 3.01 | 1.62 |
| *DNAJC7* | 8.28 | 0.72 |
| *DNAJC8* | 8.36 | 0.58 |
| *DNAJC9* | 7.64 | 0.54 |
| *ERCC1* | 8.60 | 0.57 |
| *ERCC2* | 7.07 | 0.64 |
| *ERCC3* | 7.61 | 0.47 |
| *ERCC4* | 6.63 | 0.52 |
| *ERCC5* | 5.85 | 0.63 |
| *ERCC6* | 6.43 | 0.57 |
| *ERCC8* | 7.11 | 0.48 |
| *EXO1* | 5.07 | 1.39 |
| *FEN1* | 6.56 | 0.89 |
| *LIG1* | 7.42 | 0.74 |
| *LIG3* | 7.05 | 0.89 |
| *LIG4* | 5.82 | 0.67 |
| *MGMT* | 6.50 | 1.01 |
| *MLH1* | 7.27 | 0.60 |
| *MLH3* | 7.94 | 0.58 |
| *MMS19* | 7.81 | 0.62 |
| *MPG* | 8.08 | 0.74 |
| *MRE11A* | 5.39 | 0.78 |
| *MSH2* | 8.14 | 0.72 |
| *MSH3* | 6.26 | 0.53 |
| *MSH4* | 4.12 | 1.33 |
| *MSH5* | 3.32 | 1.27 |
| *MSH6* | 7.55 | 0.64 |
| *MUTYH* | 4.96 | 0.78 |
| *NEIL1* | 3.41 | 1.36 |
| *NEIL2* | 5.94 | 0.81 |
| *NEIL3* | 6.11 | 1.18 |
| *NTHL1* | 5.85 | 0.95 |
| *OGG1* | 6.18 | 0.62 |
| *PARP1* | 8.74 | 0.70 |
| *PARP2* | 7.70 | 0.60 |
| *PARP3* | 4.38 | 1.37 |
| *PMS1* | 5.35 | 0.89 |
| *PMS2* | 8.01 | 0.81 |
| *PNKP* | 7.28 | 0.58 |
| *POLB* | 7.23 | 0.60 |
| *POLD3* | 7.15 | 0.75 |
| *POLL* | 5.75 | 1.41 |
| *PRKDC* | 8.20 | 0.75 |
| *RAD18* | 6.30 | 0.90 |
| *RAD21* | 8.67 | 0.77 |
| *RAD23A* | 8.80 | 0.76 |
| *RAD23B* | 9.67 | 0.68 |
| *RAD50* | 7.41 | 0.57 |
| *RAD51* | 5.07 | 1.03 |
| *RAD51B* | 6.94 | 0.63 |
| *RAD51C* | 6.39 | 0.55 |
| *RAD51D* | 6.57 | 0.68 |
| *RAD52* | 4.72 | 1.03 |
| *RAD54L* | 3.79 | 1.16 |
| *RFC1* | 7.83 | 0.64 |
| *RPA1* | 7.84 | 0.75 |
| *RPA3* | 4.33 | 1.09 |
| *SLK* | 8.54 | 0.61 |
| *SMUG1* | 5.97 | 0.92 |
| *TDG* | 7.91 | 0.57 |
| *TOP3A* | 7.95 | 0.82 |
| *TOP3B* | 5.45 | 1.44 |
| *TREX1* | 4.81 | 0.79 |
| *UNG* | 7.44 | 0.75 |
| *XAB2* | 7.68 | 0.59 |
| *XPA* | 6.13 | 0.64 |
| *XPC* | 6.25 | 0.75 |
| *XRCC1* | 6.21 | 0.66 |
| *XRCC2* | 8.77 | 0.71 |
| *XRCC3* | 6.05 | 0.69 |
| *XRCC4* | 5.11 | 0.71 |
| *XRCC5* | 5.92 | 1.12 |
| *XRCC6* | 4.28 | 1.06 |
| *XRCC6BP1* | 4.04 | 1.40 |
