# Supplementary material for: A DNA damage repair gene‐associated signature predicts responses of patients with advanced soft‐tissue sarcoma to treatment with trabectedin
Source: Mol Oncol. 2021 Jun 30;15(12):3691–705. doi: 10.1002/1878-0261.12996 (PMC8637557; doi:10.1002/1878-0261.12996)
Supplement: Supplementary file 5 — Table S2. Gene significantly expressed between L‐sarcoma and non‐L‐sarcomas and between grade 3 tumors vs grade 1 + 2 tumors. [file MOL2-15-3691-s008.docx]

Supplementary Table S2. Gene significantly expressed between L-sarcoma and non-L-sarcomas and between grade 3 tumors vs grade 1+2 tumors.

| Gene | Clinical factor | Mean Log2 ± SD | P |
| --- | --- | --- | --- |
| *APEX2* | Grade 1-2:  Grade 3: | 6.1 ± 1.2  6.6 ± 0.5 | 0.002 |
| *ATM* | Grade 1-2:  Grade 3: | 7.8 ± 0.6  7.5 ± 0.7 | 0.003 |
| *BRIP1* | Grade 1-2:  Grade 3: | 6.7 ± 1.2  7.0 ± 1.3 | 0.047 |
|  | L-sarcomas:  Non-L-sarcomas: | 7.1 ± 1.3  6.7 ± 1.0 | 0.039 |
| *DDB2* | L-sarcomas:  Non-L-sarcomas: | 5.3 ± 0.6  5.6 ± 0.6 | 0.019 |
| *DNAJA1* | L-sarcomas:  Non-L-sarcomas: | 9.6 ± 0.5  9.9 ± 0.5 | 0.031 |
| *DNAJA2* | L-sarcomas:  Non-L-sarcomas: | 5.6 ± 1.0  6.0 ± 0.8 | 0.005 |
| *DNAJB2* | L-sarcomas:  Non-L-sarcomas: | 7.3 ± 0.6  7.7 ± 0.8 | 0.007 |
| *DNAJB5* | L-sarcomas:  Non-L-sarcomas: | 6.2 ± 0.9  6.9 ± 1.3 | 0.003 |
| *DNAJC1* | Grade 1-2:  Grade 3: | 7.9 ± 0.7  8.1 ± 0.6 | 0.035 |
| *DNAJC10* | L-sarcomas:  Non-L-sarcomas: | 8.3 ± 0.7  8.7 ± 0.7 | 0.001 |
| *DNAJC11* | Grade 1-2:  Grade 3: | 7.1 ± 0.7  7.5 ± 0.6 | 0.015 |
| *DNAJC14* | L-sarcomas:  Non-L-sarcomas: | 6.4 ± 0.7  6.7 ± 0.7 | < 0.001 |
| *DNAJC16* | L-sarcomas:  Non-L-sarcomas: | 5.2 ± 0.6  5.5 ± 1.1 | 0.023 |
| *ERCC3* | L-sarcomas:  Non-L-sarcomas: | 7.5 ± 0.5  7.7 ± 0.4 | 0.004 |
| *ERCC6* | L-sarcomas:  Non-L-sarcomas: | 6.3 ± 0.6  6.5 ± 0.6 | 0.046 |
| *EXO1* | Grade 1-2:  Grade 3: | 4.6 ± 1.5  5.3 ± 1.3 | 0.004 |
| *FEN1* | Grade 1-2:  Grade 3: | 6.2 ± 1.0  6.8 ± 0.8 | 0.001 |
| *MMS19* | L-sarcomas:  Non-L-sarcomas: | 7.7 ± 0.5  8.0 ± 0.7 | 0.002 |
| *MSH2* | Grade 1-2:  Grade 3: | 8.0 ± 0.7  8.3 ± 0.7 | 0.011 |
|  | L-sarcomas:  Non-L-sarcomas: | 8.0 ± 07  8.3 ± 0.7 | 0.042 |
| *MSH6* | Grade 1-2:  Grade 3: | 7.3 ± 0.6  7.7 ± 0.7 | < 0.001 |
| *NEIL2* | Grade 1-2:  Grade 3: | 5.7 ± 0.8  6.1 ± 0.8 | 0.050 |
| *NEIL3* | Grade 1-2:  Grade 3: | 5.7 ± 1.3  6.6 ± 1.1 | 0.004 |
| *PMS2* | L-sarcomas:  Non-L-sarcomas: | 7.9 ± 0.8  8.2 ± 0.9 | 0.013 |
| *PRKDC* | Grade 1-2:  Grade 3: | 8.0 ± 0.6  8.4 ± 0.8 | 0.008 |
| *RAD21* | Grade 1-2:  Grade 3: | 8.5 ± 0.7  8.8 ±0.8 | 0.042 |
| *RAD51* | Grade 1-2:  Grade 3: | 4.7 ± 1.3  5.3 ± 0.8 | 0.001 |
| *RAD54L* | Grade 1-2:  Grade 3: | 3.4 ± 1.3  4.0 ± 1.0 | 0.034 |
| *RPA3* | Grade 1-2:  Grade 3: | 4.1 ± 1.0  4.4 ± 1.2 | 0.038 |
| *SMUG1* | L-sarcomas:  Non-L-sarcomas: | 5.8 ± 1.0  6.2 ± 0.8 | 0.020 |
| *TDG* | L-sarcomas:  Non-L-sarcomas: | 7.9 ± 0.6  8.0 ± 0.5 | 0.045 |
| *TREX1* | Grade 1-2:  Grade 3: | 4.6 ± 0.9  4.9 ± 0.7 | 0.012 |
| *XPC* | Grade 1-2:  Grade 3: | 6.4 ± 0.7  6.2 ± 0.8 | 0.046 |
| *XRCC3* | Grade 1-2:  Grade 3: | 5.9 ± 0.6  6.2 ± 0.7 | 0.020 |
| *XRCC5* | L-sarcomas:  Non-L-sarcomas: | 5.8 ± 1.1  6.1 ± 1.1 | 0.014 |
