# Supplementary material for: A DNA damage repair gene‐associated signature predicts responses of patients with advanced soft‐tissue sarcoma to treatment with trabectedin
Source: Mol Oncol. 2021 Jun 30;15(12):3691–705. doi: 10.1002/1878-0261.12996 (PMC8637557; doi:10.1002/1878-0261.12996)
Supplement: Supplementary file 6 — Table S3. Univariate analysis of DNA damage repair‐related genes with impact in progression‐free survival to trabectedin line. [file MOL2-15-3691-s003.docx]

Supplementary Table S3. Univariate analysis of DNA damage repair-related genes with impact in progression-free survival to trabectedin-line

| Factor | PFS (95% CI) | p | OS (95% CI) | p |
| --- | --- | --- | --- | --- |
| *ATM*   - < 7.51 - ≥ 7.51 | 3.0 (2.3-3.8)  6.3 (3.2-9.3) | 0.001 | 9.2 (3.1-15.3)  17.3 (10.2-24.5) | 0.125 |
| *CCNH*   - < 3.79 - ≥ 3.79 | 2.5 (1.9-3.0)  7.9 (4.8-11.0) | <0.001 | 8.6 (4.8-12.5)  17.8 (13.8-21.8) | 0.040 |
| *DDB2*   - < 5.49 - ≥ 5.49 | 2.5 (1.8-3.1)  6.4 (3.6-9.2) | 0.002 | 7.2 (4.1-10.4)  17.4 (14.5-20.4) | 0.024 |
| *DNAJB11*   - < 8.24 - ≥ 8.24 | 8.2 (5.2-11.1)  2.5 (2.3-2.7) | <0.001 | 17.9 (15.8-20.1)  7.4 (3.3-11.5) | 0.002 |
| *DNAJB14*   - < 7.39 - ≥ 7.39 | 3.3 (1.2-5.4)  3.8 (2.6-5.1) | 0.995 | 13.1 (6.2-20.1)  11.8 (4.3-19.3) | 0.765 |
| *DNAJC11*   - < 7.31 - ≥ 7.31 | 5.2 (2.7-7.7)  3.0 (2.2-3.9) | 0.293 | 17.8 (15.8-19.7)  8.2 (4.3-12.2) | 0.106 |
| *MUTYH*   - < 5.02 - ≥ 5.02 | 2.5 (2.0-2.9)  6.1 (4.6-7.5) | 0.007 | 9.5 (5.0-14.0)  17.4 (12.7-22.2) | 0.008 |
| *NEIL1*   - < 3.41 - ≥ 3.41 | 2.8 (2.2-3.5)  5.1 (3.2-7.0) | 0.103 | 11.8 (5.4-18.2)  15.6 (7.7-23.4) | 0.223 |
| *PARP1*   - < 8.71 - ≥ 8.71 | 6.4 (4.0-8.8)  2.5 (1.8-3.1) | <0.001 | 17.9 (15.8-20.0)  7.4 (3.5-11.2) | 0.001 |
| *PARP3*   - < 4.39 - ≥ 4.39 | 2.8 (2.1-3.6)  7.7 (4.6-1.9) | <0.001 | 7.3 (3.9-10.7)  17.5 (14.0-21.0) | 0.032 |
| *PMS1*   - < 5.27 - ≥ 5.27 | 3.4 (2.6-4.2)  4.1 (1.5-6.7) | 0.423 | 13.1 (6.9-19.3)  13.1 (4.6-21.7) | 0.984 |
| *POLL*   - < 5.53 - ≥ 5.53 | 3.2 (2.1-4.4)  3.9 (2.0-5.7) | 0.330 | 11.8 (5.5-18.0)  17.3 (10.6-24.0) | 0.513 |
| *PRKDC*   - < 8.15 - ≥ 8.15 | 5.2 (2.4-8.0)  3.2 (2.2-4.2) | 0.094 | 17.0 (11.9-22.2)  9.2 (4.6-13.8) | 0.402 |
| *RAD23B*   - < 9.68 - ≥ 9.68 | 7.3 (3.9-10.7)  2.7 (2.0-3.4) | <0.001 | 17.8 (15.7-19.9)  8.1 (2.8-13.3) | 0.043 |
| *RAD52*   - < 4.63 - ≥ 4.63 | 3.0 (2.2-3.8)  5.6 (1.9-9.2) | 0.005 | 9.5 (4.3-14.7)  17.0 (11.9-22.2) | 0.241 |
| *TDG*   - < 7.96 - ≥ 7.96 | 6.1 (3.8-8.4)  3.0 (2.2-3.8) | 0.001 | 17.9 (8.1-27.7)  8.2 (2.7-13.8) | 0.001 |
| *TOP3A*   - < 7.85 - ≥ 7.85 | 6.3 (3.1-9.4)  3.0 (2.2-3.7) | 0.006 | 18.0 (13.3-22.8)  8.1 (3.4-12.7) | 0.025 |
| *TOP3B*   - < 5.32 - ≥ 5.32 | 3.4 (2.7-4.1)  4.4 (1.8-6.9) | 0.639 | 16.4 (10.5-22.3)  11.3 (5.6-17.1) | 0.360 |
| *XPC*   - < 6.21 - ≥ 6.21 | 3.2 (2.7-3.7)  4.9 (2.3-7.5) | 0.105 | 9.2 (4.2-14.2)  17.4 (12.8-22.0) | 0.223 |
| *XRCC5*   - < 5.85 - ≥ 5.85 | 3.8 (2.5-5.1)  3.5 (1.5-5.4) | 0.567 | 17.0 (11.6-22.5)  10.2 (6.6-13.9) | 0.362 |
| *BRCA1*   - < 5.56 - ≥ 5.56 | 6.1 (2.5-9.7)  2.5 (1.6-3.3) | 0.001 | 17.8 (14.6-21.0)  8.1 (4.2-12.0) | 0.018 |
| *ERCC1*   - < 8.58 - ≥ 8.58 | 5.2 (2.5-8.0)  3.0 (2.2-3.9) | 0.084 | 14.4 (10.2-18.6)  10.2 (5.1-15.3) | 0.384 |
| *ERCC5*   - < 5.93 - ≥ 5.93 | 3.2 (2.3-4.1)  4.4 (2.5-4.5) | 0.182 | 7.4 (2.0-12.7)  17.4 (12.4-22.4) | 0.258 |
