# Supplementary material for: A DNA damage repair gene‐associated signature predicts responses of patients with advanced soft‐tissue sarcoma to treatment with trabectedin
Source: Mol Oncol. 2021 Jun 30;15(12):3691–705. doi: 10.1002/1878-0261.12996 (PMC8637557; doi:10.1002/1878-0261.12996)
Supplement: Supplementary file 7 — Table S4. Differential gene expression according to objective response. [file MOL2-15-3691-s010.docx]

Supplementary Table S4. Differential gene expression according to objective response

|  | logFC | P-Value | FDR |
| --- | --- | --- | --- |
| *MGMT* | 0.524 | 0.005 | 0.365 |
| *XRCC2* | 0.389 | 0.006 | 0.365 |
| *UNG* | 0.395 | 0.011 | 0.411 |
| *DNAJC10* | -0.336 | 0.014 | 0.411 |
| *DNAJC3* | -0.287 | 0.026 | 0.593 |
| *SLK* | -0.269 | 0.037 | 0.593 |
| *DNAJC13* | -0.238 | 0.037 | 0.593 |
| *ATR* | 0.213 | 0.043 | 0.593 |
| *RAD51D* | -0.275 | 0.045 | 0.593 |

FC: fold change; FDR: false discovery rate. A negative fold change means that the gene is overexpressed in cases with stable disease or progression disease.
