# Supplementary material for: A DNA damage repair gene‐associated signature predicts responses of patients with advanced soft‐tissue sarcoma to treatment with trabectedin
Source: Mol Oncol. 2021 Jun 30;15(12):3691–705. doi: 10.1002/1878-0261.12996 (PMC8637557; doi:10.1002/1878-0261.12996)
Supplement: Supplementary file 8 — Table S5. Differential gene expression according to histologic subtype grouping. [file MOL2-15-3691-s011.docx]

Supplementary Table S5. Differential gene expression according to histologic subtype grouping

|  | logFC | P-Value | FDR |
| --- | --- | --- | --- |
| *DNAJB5* | 0.973 | 0.000 | 0.001 |
| *DNAJC16* | -0.903 | 0.000 | 0.007 |
| *XRCC6BP1* | 1.115 | 0.000 | 0.007 |
| *MMS19* | -0.410 | 0.000 | 0.012 |
| *DNAJC10* | -0.397 | 0.001 | 0.012 |
| *DNAJB2* | -0.428 | 0.001 | 0.012 |
| *DNAJC7* | -0.538 | 0.002 | 0.029 |
| *PMS1* | 0.636 | 0.002 | 0.029 |
| *DNAJA2* | -0.418 | 0.002 | 0.032 |
| *BRIP1* | 0.632 | 0.003 | 0.037 |
| *PMS2* | -0.395 | 0.006 | 0.060 |
| *XRCC2* | 0.330 | 0.010 | 0.088 |
| *ERCC3* | -0.213 | 0.010 | 0.088 |
| *PARP3* | 0.834 | 0.011 | 0.089 |
| *ATM* | -0.337 | 0.011 | 0.089 |
| *XPC* | -0.463 | 0.012 | 0.092 |
| *NEIL2* | -0.405 | 0.016 | 0.108 |
| *POLD3* | 0.349 | 0.017 | 0.108 |
| *DDB2* | -0.261 | 0.019 | 0.108 |
| *DNAJC8* | -0.297 | 0.019 | 0.108 |
| *ERCC1* | -0.236 | 0.019 | 0.108 |
| *MLH3* | -0.235 | 0.024 | 0.130 |
| *DNAJC14* | -0.306 | 0.026 | 0.133 |
| *DNAJB14* | -0.320 | 0.032 | 0.156 |
| *ERCC6* | -0.210 | 0.034 | 0.162 |
| *SMUG1* | -0.320 | 0.040 | 0.179 |
| *RAD23A* | 0.268 | 0.041 | 0.179 |
| *PARP1* | -0.251 | 0.045 | 0.190 |

FC: fold change; FDR: false discovery rate. A negative fold change means that the gene is overexpressed in non-L-sarcomas.
