# Supplementary material for: A DNA damage repair gene‐associated signature predicts responses of patients with advanced soft‐tissue sarcoma to treatment with trabectedin
Source: Mol Oncol. 2021 Jun 30;15(12):3691–705. doi: 10.1002/1878-0261.12996 (PMC8637557; doi:10.1002/1878-0261.12996)
Supplement: Supplementary file 9 — Table S6. Differential gene expression according to tumor location. [file MOL2-15-3691-s002.docx]

Supplementary Table S6. Differential gene expression according to tumor location

|  | logFC | P-Value | FDR |
| --- | --- | --- | --- |
| *ATM* | -0.588 | <0.001 | 0.005 |
| *PMS1* | -0.809 | <0.001 | 0.017 |
| *MPG* | -0.480 | 0.001 | 0.026 |
| *POLL* | -1.470 | 0.001 | 0.026 |
| *DNAJB2* | -0.442 | 0.001 | 0.026 |
| *RPA1* | 0.427 | 0.002 | 0.041 |
| *DNAJC5* | -0.503 | 0.004 | 0.072 |
| *XRCC5* | -0.874 | 0.005 | 0.072 |
| *TOP3B* | -1.297 | 0.006 | 0.072 |
| *PARP1* | 0.360 | 0.006 | 0.075 |
| *RAD21* | 0.379 | 0.008 | 0.078 |
| *POLD3* | 0.405 | 0.008 | 0.078 |
| *PARP3* | -0.995 | 0.009 | 0.079 |
| *NEIL3* | 0.513 | 0.010 | 0.079 |
| *DNAJC4* | -0.309 | 0.010 | 0.079 |
| *DNAJB8* | -0.897 | 0.011 | 0.079 |
| *PMS2* | -0.391 | 0.012 | 0.083 |
| *DNAJC7* | -0.475 | 0.013 | 0.084 |
| *RAD51D* | 0.297 | 0.016 | 0.089 |
| *BRIP1* | 0.536 | 0.016 | 0.089 |
| *TDG* | 0.266 | 0.016 | 0.089 |
| *DNAJC15* | -0.466 | 0.017 | 0.092 |
| *MLH3* | -0.266 | 0.018 | 0.092 |
| *RAD52* | -0.562 | 0.028 | 0.137 |
| *MRE11A* | -0.272 | 0.039 | 0.185 |
| *MSH5* | -0.633 | 0.046 | 0.205 |
| *DNAJC16* | -0.507 | 0.048 | 0.205 |
| *ERCC1* | -0.216 | 0.049 | 0.205 |

FC: fold change; FDR: false discovery rate. A negative fold change means that the gene is overexpressed in somatic cases
