# Supplementary material for: A DNA damage repair gene‐associated signature predicts responses of patients with advanced soft‐tissue sarcoma to treatment with trabectedin
Source: Mol Oncol. 2021 Jun 30;15(12):3691–705. doi: 10.1002/1878-0261.12996 (PMC8637557; doi:10.1002/1878-0261.12996)
Supplement: Supplementary file 10 — Table S7. Univariate Cox regression analysis for the values of the gene signature (risk scores). [file MOL2-15-3691-s012.docx]

Supplementary Table S7. Univariate Cox regression analysis for the values of the gene signature (risk scores)

|  | β | HR  (95%CI) | Wald. Test | P value | Concordance |
| --- | --- | --- | --- | --- | --- |
| Gene signature | 1.31 | 3.72  (2.35-5.90) | 31.4 | 2.13e^-08^ | 0.7± 0.0301 |
