# Supplementary material for: A DNA damage repair gene‐associated signature predicts responses of patients with advanced soft‐tissue sarcoma to treatment with trabectedin
Source: Mol Oncol. 2021 Jun 30;15(12):3691–705. doi: 10.1002/1878-0261.12996 (PMC8637557; doi:10.1002/1878-0261.12996)
Supplement: Supplementary file 11 — Table S8. Multivariate analysis. [file MOL2-15-3691-s009.docx]

Supplementary Table S8 – Multivariate analysis

|  | Progression-free Survival | |
| --- | --- | --- |
| Variable | HR  (95% CI) | p |
| Grade 3 | 1.2  (0.8-1.8) | 0.461 |
| Non-L-sarcoma | 2.1  (1.4-3.2) | <0.001 |
| Visceral location | 1.7  (1.2-2.6) | 0.008 |
| High-risk group | 2.1  (1.4-3.2) | <0.001 |
