# Supplementary material for: A DNA damage repair gene‐associated signature predicts responses of patients with advanced soft‐tissue sarcoma to treatment with trabectedin
Source: Mol Oncol. 2021 Jun 30;15(12):3691–705. doi: 10.1002/1878-0261.12996 (PMC8637557; doi:10.1002/1878-0261.12996)
Supplement: Supplementary file 12 — Table S9. Correlation between risk groups and progression‐free survival of doxorubicin or gemcitabine. [file MOL2-15-3691-s004.docx]

Supplementary Table S9 – Correlation between risk groups and progression-free survival of doxorubicin or gemcitabine

|  | PFS Doxorubicin  (95% CI) | p |
| --- | --- | --- |
| *Gene Signature*   - Low-risk group (n=49) - High-risk group (n=20) | 6.1 (4.5-7.6)  3.4 (0.0-7.4) | 0.247 |
|  | PFS Gemcitabine  (95% CI) | p |
| *Gene Signature*   - Low-risk group (n=55) - High-risk group (n=22) | 4.1 (2.9-5.3)  3.7 (0.6-6.7) | 0.113 |
